# Stabilizing survival selection on presenescent expression of a sexual ornament followed by a terminal decline

**DOI:** 10.1111/jeb.12877

**Published:** 2016-04-24

**Authors:** M. J. P. Simons, M. Briga, S. Verhulst

**Affiliations:** ^1^ Department of Animal and Plant SciencesUniversity of SheffieldSheffieldUK; ^2^ Behavioural BiologyCentre for Life SciencesUniversity of GroningenGroningenThe Netherlands

**Keywords:** ageing, bird, carotenoids, colouration, demography, reliability theory of ageing, selective disappearance, sexual selection, zebra finch

## Abstract

Senescence is a decrease in functional capacity, increasing mortality rate with age. Sexual signals indicate functional capacity, because costs of ornamentation ensure signal honesty, and are therefore expected to senesce, tracking physiological deterioration and mortality. For sexual traits, mixed associations with age and positive associations with life expectancy have been reported. However, whether these associations are caused by selective disappearance and/or within‐individual senescence of sexual signals, respectively, is not known. We previously reported that zebra finches with redder bills had greater life expectancy, based on a single bill colour measurement per individual. We here extend this analysis using longitudinal data and show that this finding is attributable to terminal declines in bill redness in the year before death, with no detectable change in presenescent redness. Additionally, there was a quadratic relationship between presenescent bill colouration and survival: individuals with intermediate bill redness have maximum survival prospects. This may reflect that redder individuals overinvest in colouration and/or associated physiological changes, while below‐average bill redness probably reflects poorer phenotypic quality. Together, this pattern suggests that bill colouration is defended against physiological deterioration, because of mate attraction benefits, or that physiological deterioration is not a gradual process, but accelerates sharply prior to death. We discuss these possibilities in the context of the reliability theory of ageing and sexual selection.

## Introduction

One of the most intriguing things about life is that it will inevitably end. Almost all organisms age, and at first glance, this is a paradox. Death by ageing reduces the opportunity to reproduce and thereby reduces Darwinian fitness (Williams, 1957). The disposable soma theory (Kirkwood & Holliday, 1979; Ricklefs, 1998) explains how ageing can increase fitness, postulating that investments to increase reproduction are achieved at the expense of investment in somatic repair and maintenance. Physiological deterioration, not fully countered by somatic repair and maintenance, leads to a decline in functional capacity with age, that is senescence. On a demographic level, this results in accelerating (intrinsic) mortality with age (Ricklefs, 2010). Mortality risk is therefore predicted to be closely matched by deterioration of physiological parameters, that is ‘condition’ (Ricklefs, 2010). In other words, physiological parameters directly related to increased mortality risk are predicted to senesce in concordance with demographic increases in mortality rate.

The correlation between age‐specific declines in reproductive performance – a measure of condition – and mortality rate varies widely between species, however, suggesting that physiological markers of performance need not always track mortality rate (Burger & Promislow, 2006; Bouwhuis *et al*., 2012). Thus, alternatively, individuals may maintain their physiological variables at a similar level until death, when intrinsic causes of death are of a catastrophic nature (Ricklefs, 2010; Nussey *et al*., 2011). Prior to death, this may result in rapid physiological declines – terminal declines – apparent in, for example, reproduction (Coulson & Fairweather, 2001; Rattiste, 2004). A different explanation of a lack of concordance between mortality senescence and the physiology measured is that the variable measured is not causally linked to mortality (Simons, 2015) or that the physiological variable is defended against gradual senescence. The short‐term reproductive benefit of investing in the maintenance of, for example, sexual attractiveness may offset the benefit of investing in other aspects of the soma, for example immune function, with longer term reproductive benefits. The fitness return of investments with long‐term benefits is reduced by the risk of extrinsic mortality (Kirkwood & Holliday, 1979; Ricklefs, 1998), and hence, physiology associated with long‐term benefits is predicted to senesce relatively sooner.

Sexual selection has resulted in exaggerated traits (Andersson & Iwasa, 1996) that can serve as sexual signals (Kokko *et al*., 2006). The signalling value of a trait increases when cheating is effectively precluded and when it reveals information about aspects of physiology that underlie phenotypic quality (Hill, 2011). We may therefore expect traits that feature in mate choice to closely follow demographic senescence, and hence be a biomarker of ageing. This expectation will however depend on the honesty of the sexual signal in question and may change if trade‐offs maintaining signal honesty shift with age. Also if the benefits and/or costs of investing in sexual ornamentation change with age, or if an investment yields strong current reproductive benefits, sexual signals could show catastrophic rather than gradual senescence.

Associations with age have been reported for a diverse array of sexual traits. Cross‐sectional studies have reported both increasing (e.g. Budden & Dickinson, 2009; Laucht & Dale, 2012) and declining (e.g. Garratt *et al*., 2011; Edler & Friedl, 2012) signal expression with age. However, relationships with age estimated from cross‐sectional analyses can be caused by selective disappearance from the population rather than reflect changes with age within individuals (van de Pol & Verhulst, 2006). Statistically separating within‐ and between‐individual variation is required to obtain unbiased estimates of changes with age within individuals, and the few studies of this kind mainly reported increased sexual signalling with age (Delhey & Kempenaers, 2006; Nussey *et al*., 2009; Judge, 2010; Val *et al*., 2010; Evans *et al*., 2011; Kervinen *et al*., 2015). It therefore seems that we still know little about the details of the expression of sexual signals in relation to ageing despite its relevance for life‐history evolution and sexual selection. Interpreting analyses that do not separate within‐ and between‐individual variation is complicated further because sexual trait expression is generally found to be positively associated with survival (meta‐analysis in Jennions *et al*., 2001). On the population level, a positive relationship between trait expression and survival can come about via terminal declines of sexual signals, variation between individuals in senescence or associations with the level of presenescent sexual signal expression (Reed *et al*., 2008).

Here, we dissect these intricate relationships between mortality and sexual signal senescence in zebra finches (*Taeniopygia guttata*) using longitudinal data, allowing us to separate between‐ and within‐individual variation. Zebra finches form stable pair‐bonds (Silcox & Evans, 1982), but re‐pair readily if a partner is lost. Extra‐pair paternity in the wild is low (Birkhead *et al*., 1990), and reproductive success depends strongly on biparental care (Royle *et al*., 2006). Sexual selection for traits that honestly indicate quality, parental care and longevity could aid in the life‐determining choice of who to mate. Male and female zebra finches exhibit bills that are a colourful orange to deep red, pigmented by carotenoids (McGraw, 2004), which have to be acquired exclusively from the diet and are associated with immunocompetence and oxidative stress state (Simons *et al*., 2012b). Male bill colour is subject to female choice, as we recently showed using meta‐analysis across 10 separate studies (Simons & Verhulst, 2011), and is positively associated with longevity (Simons *et al*., 2012a). Positive associations of bill redness of females with survival and fledging production suggest that male choice for redder females will also yield benefits (Simons & Verhulst, 2011; Simons *et al*., 2012a). One could question whether in a captive situation where food is freely accessible carotenoids are limiting. However, birds increase in colouration when supplemented with carotenoids in captivity as well, and carotenoids and carotenoid‐dependent signals are associated with physiological parameters (Simons *et al*., 2012b). Comparative evidence suggests that carotenoid acquisition can underlie honest sexual signalling (Simons *et al*., 2014a). Furthermore, carotenoid supplementation can affect later reproduction in the same captive environment we use in this study (Simons *et al*., 2014b). These considerations have led us to interpret bill colouration as an indicator of physiological state (Pérez‐Rodríguez, 2009), also in our captive environment. We therefore analysed patterns of ageing and investigated the contribution of terminal effects in bill redness and its association with mortality in both male and female zebra finches.

## Methods

### Experimental set‐up

For six consecutive years (2007–2012), we took bill colour measurements (*n* = 1200) around mid‐November each year of males (*n* = 224) and females (*n* = 220) from our population of zebra finches housed in eight unisex outdoor aviaries (L*W*H: 320*150*225 cm). Individual birds have been added multiple times to this experiment, thereby replacing individuals that died (median longevity of a zebra finch in our population is ≈ 3.7 years). This maintained the total population of birds around 200 individuals. All birds were bred within our own facility and should be considered domesticated zebra finches (for more information see: Briga & Verhulst, 2015). These birds are used in a long‐term experiment investigating the relationships between survival, a foraging costs treatment (easy or hard foraging) (Koetsier & Verhulst, 2011), and early rearing conditions (raised in small or large broods) (de Coster *et al*., 2011). In the hard foraging treatment, individual birds have to hover in front of a feeding hole to obtain seeds (tropical seed mixture, *ad libitum*), whereas in the easy condition there is a perch allowing effortless access to the seed. Small brood (2 chicks) and large broods (6 chicks) were created by cross‐fostering broods at an age of 5 days under forced pairing in individual indoor breeding cages (L*W*H: 40*80*40 cm). Cuttlebone, grit and water were provided *ad libitum,* and the birds received fortified canary food (‘eggfood’, by Bogena, Hedel, the Netherlands) in weighed portions (Koetsier & Verhulst, 2011). The birds were left undisturbed until natural death, except for blood sampling and respirometry measurements several times a year in the context of other nonexperimental studies (always in equal measure for all treatments and ages). For identification, all birds were banded with a numbered aluminium ring. The aviaries were inspected daily, and deaths recorded until the end of December 2014. In our previous study of the association of bill colour with survival, we used only one bill colour measurement and restricted ourselves to the easy foraging condition of this experiment to avoid possible unknown confounding effects (Simons *et al*., 2012a). Here, we tested the associations of the foraging treatment and early rearing conditions, and their interaction, with longitudinal bill colour measurements, as outlined below in the statistical analysis and results section. However, we did not detect any associations with the two treatments and therefore present results across the whole population of the experiment.

### Bill colour measurement

Measurements of bill colouration were taken as described previously (Simons *et al*., 2012a). In brief, bills were digitally photographed (camera: Sony DSC‐F707) with fixed camera settings and in a controlled lighting environment. Birds were manually restrained on top of a foam mould, and the top of the bill was photographed. Digital cameras can respond to light and light composition in a nonlinear fashion (Stevens *et al*., 2007). We corrected for this using a calibration set of colour patches (Munsell glossy finish collection) with known spectra obtained from the Joensuu Spectral Database (http://cs.joensuu.fi/~spectral/databases/) to generate simulated reflectance spectra from the digital images (Stigell *et al*., 2007). Bills were automatically selected from the pictures using thresholding and cluster analysis. All these selections were manually checked and corrected in the few instances when the automatic selection procedure failed. From these bills, simulated spectra were obtained and we calculated the inflection point, which is a measure of hue, using nonlinear fitting of a 4‐parameter sigmoid curve. All the above procedures were programmed and run in MATLAB software. We validated the above method with direct measurement of reflectance, using a spectrophotometer (BLK‐C‐100 spectrophotometer, SL4‐DT (Deuterium/Tungsten) light source, R600‐8‐UV‐VIS reflectance probe; StellarNet, Tampa, FL, USA), in a subset of 31 birds. Measures of hue obtained with this method and hue from the simulated spectra of digital pictures correlated strongly (*r *=* *0.96). Repeatability of our method was high (*r = *0.997), estimated by taking two pictures from the same individual in close succession (Simons *et al*., 2012a).

### Statistical analysis

We used mixed models implemented in R (R Development Core Team, 2011) to analyse variation in bill colour. In our models, we included average age across the measures of an individual and the difference in age from this average age for each measurement (Δage), to separate within‐ and between‐individual effects (van de Pol & Verhulst, 2006). The effect of Δage (centred around the average age at measurement) provides an estimate of the within‐individual slope of age against bill colour independent of selective disappearance. The term average age tests the effect of age across individuals and is thus dependent on effects of selective disappearance. In addition, we investigated terminal effects by fitting a binomial factor coding for whether an individual died a natural death in the subsequent year or not. All these models included a random effect at the intercept for each individual and a random effect of slope for Δage across individuals. Neglecting to include random‐slopes in mixed models is likely to result in erroneous conclusions (Schielzeth & Forstmeier, 2009). We included two additional random intercepts in the mixed models: the year in which measurements were taken and the birth nest (210 individual nests) of the individuals.

Within the analyses of bill hue senescence, we tested for main effects of foraging treatment and rearing brood size (and their interaction) and for interactions with the independent variables included in these models of the foraging treatment and rearing brood size (and their interaction). We selected the best model among the models that contained our hypothesized variables of interests (see result section) using a best subsets approach, that is fitting all possible variables combinations, using the MuMIn package in R, based on BIC (Bayesian information criterion). In practice, this resulted in the models that excluded terms (ΔBIC > 2.3) related to both the foraging treatment and rearing brood size.

To assess relationships of trait values with survival, we fitted right‐censored Cox proportional hazards (Survival package in R, ‘coxph’). Censored cases included birds that were still alive, died within 48 h after handling for experimentation or by accident (*n* = 34), and birds that were terminated for various welfare considerations (*n* = 12). Violations of the proportional hazards assumption were tested using the ‘cox.zph’ function and by plotting scaled Schoenfeld residual plots. No such violations were detected.

To contrast cross‐sectional population level analyses with within‐individual analyses, we also analysed survival on a yearly basis, by estimating the difference in bill hue between survivors and birds that died in the subsequent year. These estimates were summarized across years using a fixed‐effects meta‐analysis (Viechtbauer, 2010), and the associated confidence interval of the average effect corrected for the dependence within the data due to multiple measures from the sample individual (Higgins & Green, 2008). This entailed inflating the associated standard error by multiplying it by the square root of the fraction of the dependent sample size (the number of measurements) over the independent sample size (the number of unique individuals). We investigated both male and female bill colouration, and all models were tested separately for each sex.

## Results

### Mortality and bill colour on the population level

**Figure 1:**
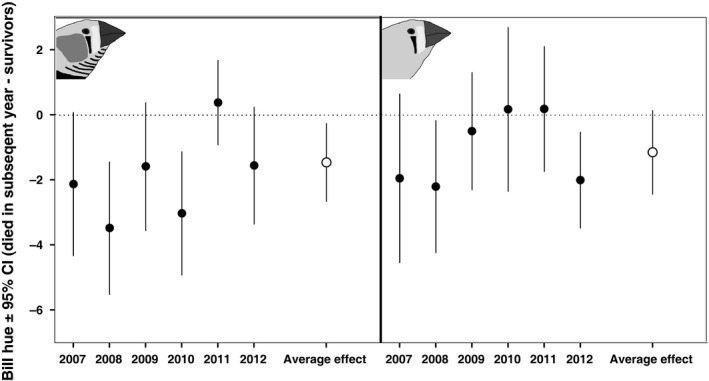
Estimated bill hue difference between the individuals that died in the subsequent year and those that survived for each year of the study (filled circles), and the average effect across the years of measurements (open circles). In both males (left panel) and females (right panel), lower bill hue was associated with mortality in the subsequent year. The error bars indicate 95% confidence intervals, and the dotted horizontal line at zero indicates no difference in bill hue between individuals that died in the subsequent year and the survivors.

To contrast the results of a cross‐sectional analysis with the within‐individual analyses that follow, we first tested for the six separate years of our study whether the individuals that died in the subsequent year following our measurement had lower bill hues (Fig. 1). We find that for both males (*z* = −2.40, *P* = 0.016) and females (*z* = −1.73, *P* = 0.08) lower bill hues are associated with lower survival in the subsequent year (Fig. 1).

### Within‐ and between‐individual associations with age

We first investigated the dependency of bill colour on age separating within‐ and between‐individual effects (Table 1A), which included average age (between‐individual effect) and Δage (within‐individual effect). The necessity to examine between and within‐individual effects of age simultaneously was evident from the result; because for males, we found a significant decrease in bill hue with age within individuals, but a significant positive slope between individuals. This indicates selective disappearance of individuals with low bill hues from the population, causing an increase of average bill hue with age. Within females, the same pattern emerged, but is not significant, but note that the standard errors of the Δage and average age estimate do not overlap (Table 1A), which is indicative of significant selective disappearance in females as well (van de Pol & Verhulst, 2006).

**Table 1:** (A) Bill hue modelled as a function of within‐ (Δage) and between‐individual (average age) effects of age. (B) The model of bill hue presented in (A), but extended with a factor coding for the last measurement prior to natural death (= 1 when it died in the subsequent year, = 0 when it did not). Note that measurements in the year prior to censoring are excluded from this data set. (C) The model presented in (B) with the selection from the data set including only individuals that were measured at least three times and died a natural death

	Term	Estimate (±SE)	*P*
A			
Males (*n* = 224 birds, 616 measurements)	Δage	−0.59 (0.18)	0.0015
	Average age	0.62 (0.24)	0.011
Females (*n* = 220 birds, 584 measurements)	Δage	−0.41 (0.22)	0.06
	Average age	0.26 (0.28)	0.36
B			
Males (*n* = 217 birds, 591 measurements)	Δage	−0.24 (0.21)	0.26
	Average age	0.43 (0.25)	0.086
	Died in subsequent year	−1.44 (0.40)	0.0004
Females (*n* = 213 birds, 561 measurements)	Δage	−0.09 (0.27)	0.74
	Average age	0.17 (0.29)	0.56
	Died in subsequent year	−0.79 (0.44)	0.074
C			
Males (*n* = 63 birds, 257 measurements)	Δage	0.052 (0.38)	0.89
	Average age	0.025 (0.59)	0.96
	Died in subsequent year	−2.09 (0.65)	0.0015
Females (*n* = 72 birds, 292 measurements)	Δage	0.19 (0.44)	0.67
	Average age	0.29 (0.67)	0.66
	Died in subsequent year	−0.11 (0.61)	0.86

### Terminal effects

**Figure 2:**
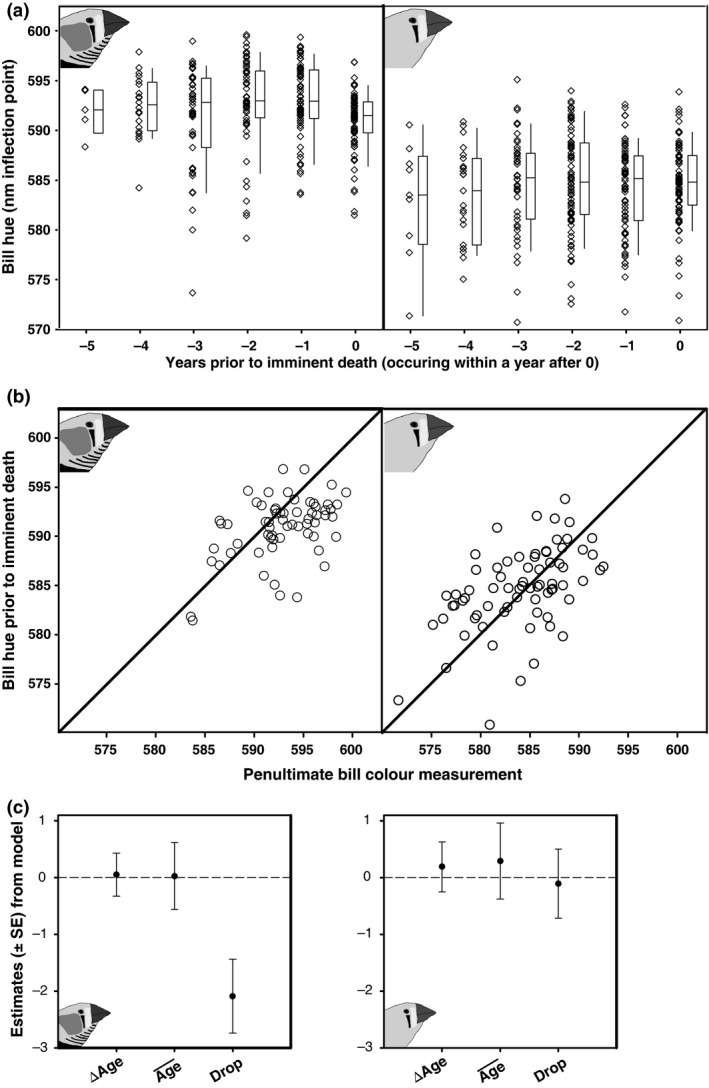
Longitudinal patterns in bill hue. (a) Bill hue drops in the year prior to imminent death in males (left panel) with no evidence for senescence in both sexes prior to this point. Data are raw data and box plots from a subset of individuals that all died a natural death and were measured for three or more years (see Table 1C). (b) Drops in bill hue visualized on the individual level. Presenescent bill hue, measured in the year penultimate to the year of death, is plotted against the last measurement prior to death. Outside this mixed model context (Table 1C), matched‐pairs *t*‐tests resulted in the same conclusions (males: *t*_62_ = −4.26, *P* < 0.0001; females: *t*_71_ = 0.99, *P* = 0.32). (c) Estimates of Δage, average age and terminal declines in the year prior to imminent death from the model presented in Table 1C.

Next, we investigated terminal effects, by adding a factor indicating whether the bird died in the subsequent year following the bill colour measurement or not (Table 1B). We omitted the last bill colour measurements of birds that were censored (see Methods) from this analysis, because we do not know whether these birds would have died a natural death in the year following the last measurement or not. In both sexes, death was preceded by a drop in bill hue, although note that this effect was significant in males, but 45% smaller in females and statistically only a trend (Table 1B). Because in these models some individuals are only measured once or twice, this causes Δage and ‘died in subsequent year’ to code for essentially the same change in these individuals, not allowing the model to separate the two. Moreover, not all individuals in this set have died yet, also potentially biasing the results, because in these individuals the terminal effect cannot be estimated. Therefore, we also tested the terminal effect in a truncated data set, including only birds for which three or more measurements were available and that had died (Table 1C). Also in this set we find, although only for males, that imminent death is accompanied by a drop in bill hue (Fig. 2). In both sexes, the parameter estimate of Δage is reduced in magnitude and becomes nonsignificant when we include the terminal effect in the models, suggesting that bill hue does not change prior to the terminal decline that precedes death. This also suggests that there is no selective disappearance with respect to bill colouration other than through the decline in colouration associated with imminent death.

We refrained from including quadratic age terms, because in our data set the number of individuals with three measurements or more is limited. Moreover, the inclusion of a quadratic age term would complicate the independent estimation of a terminal effect and would require further restriction of the data set (from data in Table 1C). However, it is not unusual for ornamentation to increase with age early in life, and to test for such an effect we investigated whether birds in their first year of life had lower bill hue. By adding ‘first year’ as factor to the model presented in Table 1B, but found no evidence for such an effect in either females (estimate −0.14 ± 0.52, *P* = 0.79) or males (estimate −0.15 ± 0.48, *P* = 0.74). To scale the magnitude of the terminal decline in bill hue, we calculated the repeatability of presenescent bill hue (males, *r* = 0.36 ± 0.08, *P* < 0.0001; females, *r *=* *0.50 ± 0.07, *P* < 0.0001) and the standard deviation of the penultimate measurement prior to death (males, SD = 4.0; females, SD = 4.6). The terminal decline we detect in males thus reduced bill hue by 0.53 SD (Table 1C), and presenescent bill colouration was repeatable between years.

### Association between presenescent bill hue and survival

**Figure 3:**
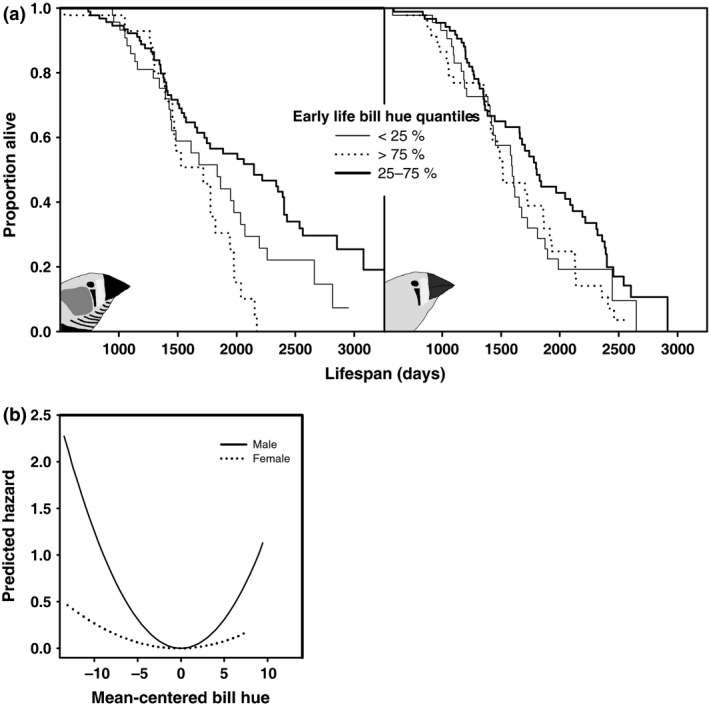
Presenescent bill hue and survival. (a) Survival patterns of subgroups that differed in presenescent bill hue (the yearly bill hue measurement prior to the last measurement before natural death or censoring). Plotted are quantiles but note that the proportional hazard models (Table 2) were based on continuous data. Also note that the plot only contains individuals for which presenescent bill hue could be assessed and that bill colouration measurements started in adulthood (>160 days), explaining the high survival at young ages in the plot. Therefore, the *x*‐axis of the plot was truncated to the shortest lifespan observed in the set for visualization purposes. (b) Predicted quadratic relationships with bill hue and hazard of death from the proportional hazard models in which bill hue was entered as continuous variable (Table 2), plotted for the full range of the underlying data. Intermediate bill hues are associated with higher survival.

Given that bill hue did not systematically change with age before a terminal decline preceding death, a distinction can be made between presenescent and senescent bill hue. To examine whether presenescent bill hue is associated with survival, we used the last measurement prior to the year that was followed by death or censoring in the subsequent year, corrected for measurement year in a mixed model. We only included one data point per individual instead of an average, to avoid regression to the mean biasing our estimates (the longest living individuals would have more measurements, and hence through stochastic effects an average closer to the population mean); however, associations with survival using an estimated average presenescent bill hue per individual were very similar (data not shown). We entered presenescent bill hue values (mean centred per sex) into a Cox proportional hazards survival analysis in which we tested both linear and quadratic effects. We found that the data were best described by the quadratic term of bill hue alone in males (Table 2), indicating better survival of individuals with a bill hue close to the average (Fig. 3). In females, this pattern was similar in shape but smaller in magnitude and not statistically significant (Table 2, Fig. 3). The linear term of bill hue was small for both males (estimate: 0.06 ± 0.034, *P* = 0.06) and females (estimate: 0.023 ± 0.024, *P* = 0.35). Note that in the models that did include the linear term of bill hue, the quadratic term of bill hue was also significant in males (*P* = 0.0002) and again not significant in females (*P* = 0.22). To test whether this pattern is driven by stronger directional selection at one or the other side of this optimum, analyses of the associations with survival in the least red and reddest half of the data were conducted. We detected significant negative survival selection at both ends of the intermediate bill hue in males (Table 2). These results indicate that mortality is lowest for individuals with presenescent bill hue close to the average (Fig. 3B) and increases when presenescent bill hue deviates more from the average in either direction (Table 2).

**Table 2:** Proportional hazard models estimating the relationship between presenescent bill hue and survival prospects. The full sets contained 180 females (64 censored), 184 males (83 censored). Note that the significance of the quadratic effects reported here is not dependent on the exclusion of the linear term from the models (see text)

	Term	Estimate (±SE)	*P*
Males	Presenescent bill hue^2^	0.0127 (0.004)	0.0018
Females	Presenescent bill hue^2^	0.0028 (0.0031)	0.38
Males (only least red half of data)	Presenescent bill hue^2^	0.011 (0.0048)	0.022
	Presenescent bill hue	−0.121 (0.054)	0.026
Males (only reddest half of data)	Presenescent bill hue^2^	0.030 (0.011)	0.002
	Presenescent bill hue	0.238 (0.090)	0.008
Females (only least red half of data)	Presenescent bill hue^2^	0.0003 (0.0038)	0.93.
	Presenescent bill hue	−0.026 (0.046)	0.58
Females (only reddest half of data)	Presenescent bill hue^2^	0.012 (0.0069)	0.086
	Presenescent bill hue	0.118 (0.060)	0.048

## Discussion

**Figure 4:**
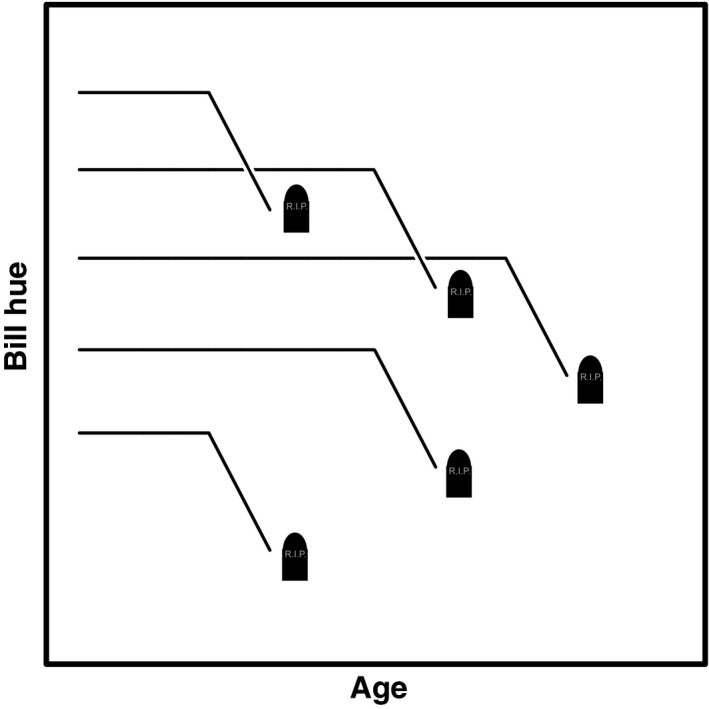
Schematic representation of the main results. The separate lines depict hypothetical individuals with different bill hues and lifespans. Bill hue drops prior to imminent death (as indicated by the gravestones). There is no evidence of senescence before this drop. Individuals with intermediate bill hue in early life (before the drop in bill hue) survive longest. Note that these associations were stronger and statistically significant within males and weaker but similar in direction within females.

In summary, we find that bill hue drops sharply when death is imminent without prior signs of improvement or senescence and that individuals with average presenescent bill hue have the best survival prospects (schematic overview in Fig. 4). Associations within females are in the same direction as in males, but weaker and hence not statistically significant in all analyses, despite a significant association between bill hue and survival also in females (Fig. 1; Simons *et al*., 2012a). We therefore tentatively conclude that qualitatively the same pattern holds in females as in males, but less strongly, and therefore, more data are required to find statistically significant results. The positive associations of bill colour with survival we reported earlier (Simons *et al*., 2012a) can thus be attributed to the combined effect of lowered survival of individuals that have low presenescent bill hue and the drop in bill hue associated with imminent death.

Positive associations between ornament expression and survival have often been reported (Jennions *et al*., 2001), but it remains to be investigated whether the underlying pattern of negligible senescence, a terminal decline and stabilizing survival selection we find in our study is also general. We know of only one other report of a similar pattern: Common guillemots (*Uria aalge*) show declines in breeding success in the last years prior to death and presenescent breeding success shows a quadratic relationship with reproductive lifespan, with longest reproductive lifespans for the individuals with average early‐life reproductive output (Reed *et al*., 2008). The multiple steps of analysis required to arrive at our and Reed *et al*.'s conclusions may be a reason why similar results have not been reported in other species. The generality of this pattern for fitness linked traits therefore warrants more study.

Our results have implications for mate choice, because they indicate that declines in bill hue signal imminent death and hence potential mates with low bill hue should be avoided. This strategy would yield benefits, because individuals with low bill hue have lowered short‐term (Figs 1 and 2) and long‐term (Fig. 3) survival prospects, which put breeding attempts at risk because zebra finches depend strongly on biparental care (Royle *et al*., 2006) and re‐mating can be costly (Ens *et al*., 1993; van de Pol *et al*., 2006). Yet, the reddest individuals also suffer from reduced survival probabilities (Fig. 3). This could indicate that these reddest individuals overinvest into their ornaments and associated physiology, reducing their survival, in line with the disposable soma theory (Kirkwood & Holliday, 1979). Overinvestment into the ornament yields increased attractiveness (Simons & Verhulst, 2011), possibly because it obscures the terminal decline in bill hue to potential mates (Fig. 4). The costs of losing a mate could be a functional reason to avoid the reddest males in mate choice. Nondirectional preferences, as previously shown for mate choice by zebra finches males (Burley & Coopersmith, 1987), are a possible solution to avoid potential mates that overinvest in their ornamentation (Chenoweth *et al*., 2006). Note however that female zebra finches do prefer males artificially manipulated to display super red bills (beyond the natural range) (Burley & Coopersmith, 1987). It is tempting to speculate that the possible differences between male and female choice evolved to match differential investment into reproduction in females and males (Chenoweth *et al*., 2006). Yet, in mate choice in general, and also in the zebra finch (Simons & Verhulst, 2011), the exact shape of preference functions is rarely tested, possibly because mate‐choice experiments are hard to do (Bell *et al*., 2009). Note that reduced survival does not need to be directly related to overinvestment in the ornament. It could also be that these reddest individuals have larger reproductive capacities and associated physiological adaptations, which may be only slightly offset by reduced survival. For instance, we have earlier reported higher fledgling production by the redder females (Simons *et al*., 2012a). Reduced survival of the individuals exhibiting the reddest presenescent bills does therefore not necessarily point to cheating, but can also represent a different life‐history strategy.

The zebra finch bill therefore provides different information at different life stages (Fig. 4). This nuance is likely not exclusive to the zebra finch bill but could be a general property of sexual signals (Candolin, 1999). Intermediate presenescent bill hue is associated with highest survival, whereas in general the most ‘yellow’ individuals survive worst because bill hue drops when death approaches. Phenotypic correlations (e.g. immunocompetence, condition, behaviour) with sexual traits (e.g. colouration) likely differ in strength and perhaps even sign between these life‐history stages and this may explain why these associations are relatively weak (Nakagawa *et al*., 2007; Simons *et al*., 2012b). Hence, we might rather expect mates to monitor bill colouration changes in their partner and use this information to decide on divorce or reproductive investment. Indeed, experimentally reducing foot colouration after pair‐bond formation of blue‐footed booby males reduced female courtship behaviour and propensity to copulate (Torres & Velando, 2003). Mate choice for a first or novel social or sexual partner is likely based on avoidance of individuals with low bill hues, and on choice for redder bills, in all likelihood driven by the expected association with reproductive capacities or the benefit of producing more attractive offspring. In the captive, single sex conditions in which the zebra finches in this study were kept these sexually selected benefits were not acting and we cannot exclude that the birds may have modulated sexual signalling accordingly. The birds could however not know that they would spent their lives without reproductive opportunities and this is probably also the reason they kept their signalling efforts up, or for reasons of intrasexual competition.

On the individual level, mortality risk is effectively tracked by terminal declines in bill hue. Yet bill hue before the terminal decline does not senesce and individuals with intermediate presenescent bill hue survive best (Figs 2, 3, 4). Prior to the terminal decline, bill hue does not signal physiological deterioration underlying mortality. This finding is also illustrated by the fact that we did not find effects of the foraging or the rearing brood size treatment on bill hue, even though these treatments do affect survival rates (M. Briga in preparation). Although we tested for confounding effects of our foraging treatment and brood size manipulation within the current data set, we cannot exclude that associations would be different under a harsher environmental manipulation or in the field. On a more positive note, compared to other work investigating relationships with sexual signalling in a control laboratory environment only, we can generalize our results further because they hold across our range of mild manipulations of environmental quality in early and adult life. Potentially harsher and more immediate manipulations of physiological state than rearing brood size and foraging treatment, like an immune challenge (Alonso‐Álvarez *et al*., 2004) and cold exposure (Eraud *et al*., 2007) have in contrast been shown to reduce zebra finch bill colouration. Bill colouration is thus likely defended against physiological deterioration, probably because of its attractiveness benefits, except when facing immediate severe physiological challenges.

Alternatively, it may be that physiological deterioration underlying senescence is not a gradual process but accelerates sharply prior to death. Indeed, fecundity in black‐legged kittiwakes (*Rissa tridactyla*), common gulls (*Larus canus*) and common guillemots (*Uria aalge*) has been found to also drop prior to imminent death (Coulson & Fairweather, 2001; Rattiste, 2004), but also more complicated terminal effects, interacting with age, on reproduction have been reported (Torres *et al*., 2011; Hammers *et al*., 2012). Yet other studies do not find these effects in, for example, great tits (*Parus major*) (Bouwhuis *et al*., 2009) and mute swans (*Cygnus olor*) (McCleery *et al*., 2008), where reproductive senescence was found to be gradual. It would be illuminating to unravel to what extent these different senescence trajectories on the demographic level are paralleled by different physiological senescence trajectories, because both the absence and the presence of such parallels would provide information on the ageing process.

Physiological markers that are correlated to mortality risk, such as telomeres, can potentially be revealing in this respect (Boonekamp *et al*., 2013; Simons, 2015). Interestingly, telomere shortening also accelerates sharply prior to imminent death in jackdaws (*Corvus monedula*) (Salomons *et al*., 2009). Telomeres are DNA/protein structures at the end of chromosomes, are sensitive to oxidative stress, decline in length with age (Riethman, 2008), and in humans behave as a biomarker of somatic redundancy (Boonekamp *et al*., 2013). Reliability theory of ageing postulates that the soma is composed of redundant units, which fail at a certain rate, and when redundancy is depleted the organism dies (Gavrilov & Gavrilova, 2001). Usually, failure rate of redundancy units is assumed to be constant (Gavrilov & Gavrilova, 2001; Boonekamp *et al*., 2013), yet this does not need to be the case (Simons *et al*., 2013). Terminal declines in physiological parameters such as telomere length, reproduction and sexual signalling shortly before death may indicate that failure rate increases shortly before death, or represent a physiological collapse when redundancy is almost exhausted. This exemplifies that research on connections between changes with age in biomarkers (Boonekamp *et al*., 2013; Simons, 2015) of physiological functioning and demographic patterns of deaths may prove highly fruitful in understanding the biology of ageing. Sexual ornaments may be excellent traits to study these connections, because of their intimate relationship with physiological state.
